# Influence of conspecifics and road noise on the properties of the advertisement call of *Phyllodytes luteolus* (Anura, Hylidae)

**DOI:** 10.1002/ece3.11261

**Published:** 2024-08-07

**Authors:** Leonardo Marques de Abreu, Vinicius Guerra, Mirco Solé

**Affiliations:** ^1^ Programa de Pós‐graduação em Ecologia e Conservação da Biodiversidade Universidade Estadual de Santa Cruz Ilhéus Bahia Brazil; ^2^ Laboratório de Herpetologia Tropical Universidade Estadual de Santa Cruz Ilhéus Bahia Brazil; ^3^ Laboratório de Herpetologia e Comportamento Animal, Instituto de Ciências Biológicas Universidade Federal de Goiás Goiânia GO Brazil; ^4^ Herpetology Section Zoologisches Forschungsmuseum Alexander Koenig Bonn Germany

**Keywords:** acoustic communication, anurans, bromeligens, mate attraction, noise pollution

## Abstract

Acoustic communication in animals can be affected by multiple biotic (intra and interspecific) and abiotic (e.g., wind and rain) natural noises. In addition, human beings produce additional novel sources of noise, which can reduce or inhibit the reception of acoustic signals by conspecifics, leading to behavioral changes. In this study, we investigated whether sound of conspecifics and road noise additively affect the acoustic parameters of the advertisement call of males of a Yellow Heart‐tongued Frog (*Phyllodytes luteolus*). We hypothesized that males that vocalize in choruses (males calling nearby) and in areas close to highways (anthropic noise) will increase their temporal and spectral acoustic parameters, respectively, to avoid acoustic signal masking. We recorded the vocalizations of 38 males in environments close (*N* = 18) to and distant (*N* = 20) from highways in different social contexts (many or few individuals calling nearby). Contrary to our expectation, the results indicated that males calling in areas close to highways had lower dominant frequency calls than those from natural areas (far from highways), and that the density of males in the chorus had no influence on the acoustic parameters. Furthermore, we found a positive relationship between body size and intensity, indicating that larger individuals can emit calls that can reach greater distances. The advertisement call of *Phyllodytes luteolus* has a high dominant frequency, with little overlap with the frequency of anthropic noises (roads), which may explain its presence and reproductive success of this species in bromeliads from urbanized areas.

## Graphical abstract

In this work, we investigated the influence of motor vehicle noise on a highway in the municipality of Ilhéus on the acoustic parameters of the advertisement call of a species of anuran with bromeligenous habits. This was the first study to investigate this effect on a species that spends its entire life in bromeliads and to present data/values of its acoustic parameters of its vocalization. Their populations are susceptible to various impacts due to their peculiar habit of living in bromeliads, in addition to occurring in restinga environments.

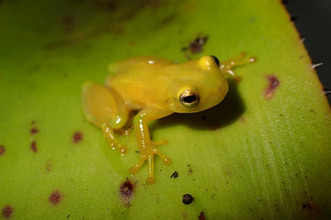

## 1 INTRODUCTION

Acoustic communication is widely used by animals to transmit information through sounds. These sounds are produced by an emitter (source) and are propagated in the environment, causing some response in the receivers (Kime et al., 2000; Wells, 2007). In environments where acoustic signals propagate, transmission can be hampered by other different sounds, causing stress, irritability, reduced fitness, in addition to being associated with other risk situations (Grenat et al., 2019; Leon et al., 2019; Troïanowski et al., 2017). There are three main types of sound noise that interfere with the transmission and detection of the species' acoustic signal: abiotic (environmental), such as the presence of winds, rains, streams and ocean tides (Caldart et al., 2016); biotic, produced by intra and interspecific individuals that can form dense social groups (Lengagne, 2008); and anthropic, which are related to acoustic pollution caused by humans, such as the flow of automobiles on highways, civil construction machinery, air transport, ships and boats (Brumm & Slabbekoorn, 2005; Cunnington & Fahrig, 2010, 2012). The interference caused by these noises can negatively influence the fitness of individuals, and consequently affect populations and communities (Barber et al., 2011; Hanna et al., 2014; Hoskin & Goosem, 2016). Over the last few decades, there has been an increase in studies on the effects of noise on the acoustic communication of organisms (Gomes et al., 2022; Grenat et al., 2019; Slabbekoorn et al., 2018).

More attention has recently been directed to anthropic noises. This type of noise alters the conditions of the acoustic environment of many habitats, creating new environmental pressures that directly affect many animals that communicate acoustically, including frogs (Barber et al., 2010; Desrochers & Proulx, 2017; Kight & Swaddle, 2011; Sabagh et al., 2017; Zaffaroni‐Caorsi et al., 2023), reptiles (Brumm & Zollinger, 2017) birds (Bermúdez‐Cuamatzin et al., 2009; Gil & Brumm, 2015; Herrera‐Montes & Aide, 2011; Slabbekoorn & Ripmeester, 2008), fishes (Popper & Hastings, 2009) and terrestrial and marine mammals (Finneran & Branstetter, 2013; Melcón et al., 2012; Moore & Clarke, 2002; Slabbekoorn et al., 2018; Stocker, 2002). Among the anthropic noises, highways are considered the biggest source of noise pollution, producing sounds with high energies concentrated in low frequencies (<5 kHz) (Warren et al., 2006). The urban expansion, and consequently the road network, not only decreases the availability of habitats but also increases the amount of human noise, causing negative effects on the transmission and reception of sound between conspecifics (Bittencourt et al., 2016; Sun & Narins, 2005), and may even reduce the chances of survival of individuals (Gomes et al., 2022; Herrera‐Montes & Aide, 2011). One of the groups of animals most affected by human noise are anuran amphibians (Zaffaroni‐Caorsi et al., 2023), which use acoustic signals as their main form of communication (Gomes et al., 2022; Wells, 2007).

During the breeding period, most anurans form dense aggregations in water bodies (Wells, 2007). Communication between frogs occurs mainly through the emission of different types of vocalizations (Toledo et al., 2015), however, the most emitted acoustic signal is the advertisement call, which has the main function of attracting reproductive partners and delimiting territories (Guerra et al., 2018; Toledo et al., 2015; Wells, 2007). The calls of conspecific individuals (and also of other species) can represent biotic sound noises that interfere in the local acoustic space. Thus, males in vocalization activity must avoid the overlapping of these acoustic signals (e.g., temporal and spectral parameters) in some way (Bittencourt et al., 2016; Herrera‐Montes & Aide, 2011). Dense choruses of males in vocalizing activity may also lead to limitations in the ability of females to choose reproductive partners (Wollerman & Wiley, 2002). However, the species show several solutions to solve the problems in the communication limitation imposed by the noises, such as, changing the temporal and spectral acoustic parameters of the calls to reduce the noise masking effect (Cunnington & Fahrig, 2010, 2012; Grenat et al., 2019).

Among the strategies used by anurans to reduce or avoid the overlap between biotic and anthropic noises on their calls, there are changes in amplitude (Halfwerk et al., 2016; Parris et al., 2009; Yi & Sheridan, 2019), frequency (Caorsi et al., 2017; Cunnington & Fahrig, 2010), duration (Zhao et al., 2021) and emission rate (Hanna et al., 2014; Kaiser & Hammers, 2009; Legett et al., 2020). These changes can be advantageous when the individuals are under external influences, since the acoustic signals indicate the physical condition of the individuals. Therefore, they must be transmitted in the best possible way in the environment (Cunnington & Fahrig, 2010; Kime et al., 2000), just as the acoustic adaptation hypothesis predicts (Goutte et al., 2018; Morton, 1975). Thus, changes in parameters of the call may indicate an adaptation in response to noise, but they may generate additional fitness costs, negatively affecting survival and reproductive success (Herrera‐Montes & Aide, 2011). It is often difficult to find evidence that suggests that changes in the calls of individuals observed in nature are caused by a single factor (Grenat et al., 2019), as variations and/or adjustments in acoustic parameters can be influenced by the environment (abiotic factors; Kime et al., 2000), size of chorus or number of the conspecifics males (social factors; Gambale & Bastos, 2014; Morais et al., 2012) and/or level of human noise (Caorsi et al., 2017). Therefore, there may be confounding factors when trying to explain changes in behavior if the study does not consider the multiple biological and environmental aspects to which individuals are exposed.

Biotic factors (body size, weight, predation and abundance of males in vocalization activity) and abiotic factors (temperature, humidity and vegetation heterogeneity) influence the anurans vocalizations in different ways. For example, body size influences the spectral structure (frequency) of the call, so that larger individuals present calls with lower frequencies (Köhler et al., 2017). Thus, acoustic signals provide reliable information about male body size (Bastos et al., 2011; Morais et al., 2012). The number of individuals in the chorus influences the intensity of the call as males increase sound pressure to promote greater attractiveness (Bastos & Haddad, 2002; Morais et al., 2012). As frogs are ectothermic animals, temperature influences the metabolic rate, reflecting changes in the temporal parameters of calls, such as duration and emission rate (Bastos & Haddad, 2002; Furtado et al., 2016). All these aspects must be considered in bioacoustics studies to avoid bias in the interpretation of results.

Recently, Zaffaroni‐Caorsi et al. (2023) carried out a literature review on the effects of anthropogenic noise on anurans, and found evidence that physiological (e.g., increased stress and suppression of immune function) and behavioral (e.g., vocalization activity) changes may occur, with consequences for sexual selection. Since human activities have impacted the behavior of amphibians in different ways, in this work we evaluated whether the call of a Yellow Heart‐tongued Frog species is affected by noise pollution produced by car traffic on highways and by the noise of conspecifics in the chorus. We hypothesized that (1) males exposed to anthropic noise (road traffic) will present a higher dominant frequency of the advertisement call to decrease or avoid signal masking, and that (2) males that vocalize in conspecific choruses with higher density of individuals will present higher values in the temporal parameters of the call (e.g., longer call duration and decrease in the interval between calls) to increase the efficiency in signal transmission (and reduce or avoid overlapping of the call) in the environment. For this, we compared the acoustic parameters of advertisement calls of males of *Phyllodytes luteolus* (Wied‐Neuwied, 1821) from natural and urban environments, and in the presence of many or few conspecific calling males. *Phyllodytes luteolus* is an excellent model organism to test these hypotheses because it is a common species, forms reproductive choruses, uses acoustic signals as the main form of communication and is found in bromeliads in natural and urban environments (Forti et al., 2017; Salles & Silva‐Soares, 2010).

## 2 MATERIALS AND METHODS

**FIGURE 1:**
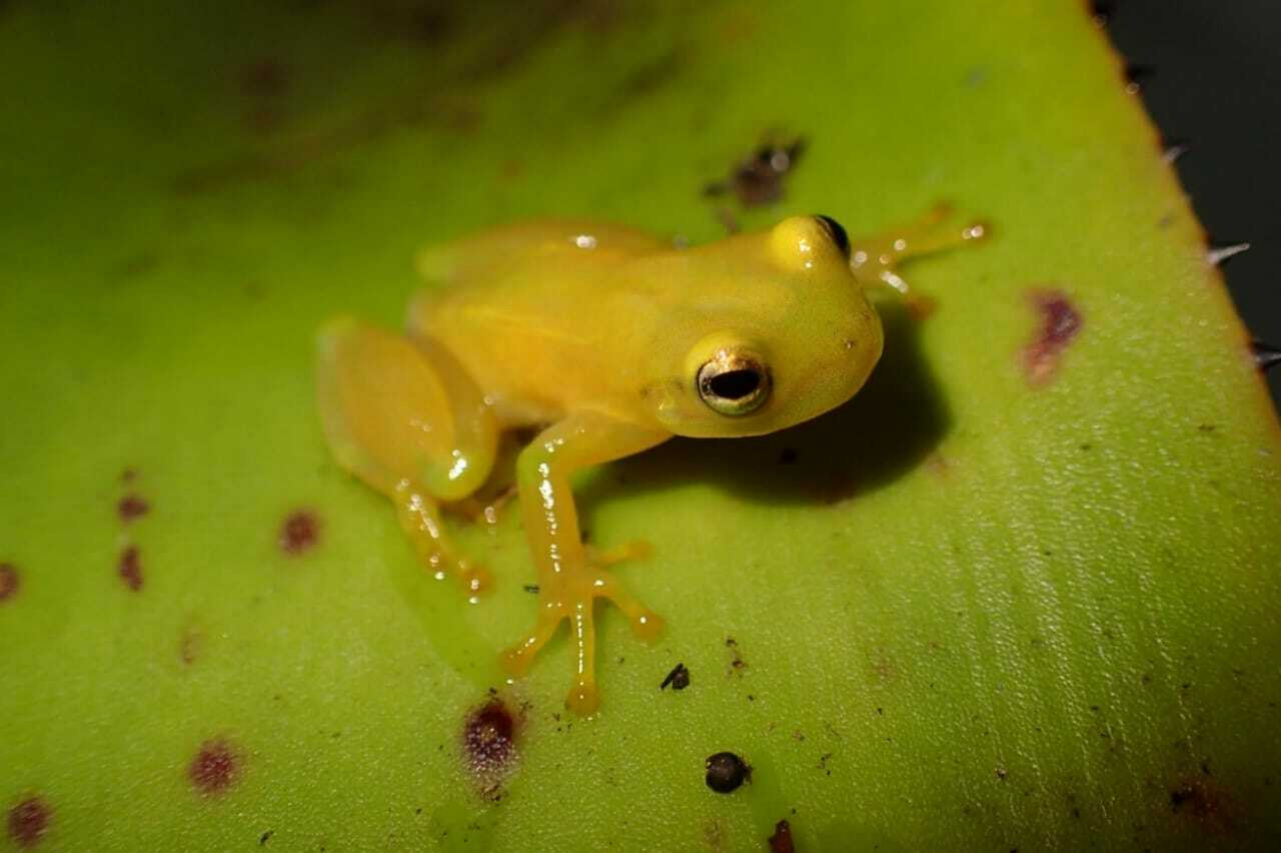
Male individual of *Phyllodytes luteolus* (Wied‐Neuwied, 1821) on the leaves of the bromeliad *Aechmea blanchetiana*. Vila do Acuípe, municipality of Ilhéus, state of Bahia, Brazil. Photo: João Victor A. Lacerda.

*Phyllodytes luteolus* (Wied‐Neuwied, 1821) (Figure 1) is a species of anuran amphibian belonging to the Hylidae family. This species spends its entire life cycle inside bromeliads and is considered to have a bromeliad habit (Peixoto, 1995). They prefer bromeliads with greater complexity of structures and when they form a network connected by several individuals of the same bromeliad species (Eterovick, 2008; Ferreira et al., 2012; Mageski et al., 2016). Reproduction is prolonged, occurring throughout the year (Ferreira et al., 2012). However, the reproductive activity of the species is greater during spring, when the climate is rainier and warmer.

The advertisement call of *P. lutelus* consists of a pulsed call, with a series of notes without frequency modulation with an average duration of 5 seconds, and dominant frequencies ranging between 2 and 4 kHz (Cruz et al., 2014; Simon & Gasparini, 2003). *Phyllodytes luteolus* males begin vocalization activity around 2 h after sunset (7 pm). The tadpoles can prey on disease vector mosquito larvae acting in the biological control of possible diseases transmitted by these organisms (Salinas et al., 2018). In addition, this species has a diet specialized in ants with pharmacological potential (Solé & Loebmann, 2017).

**FIGURE 2:**
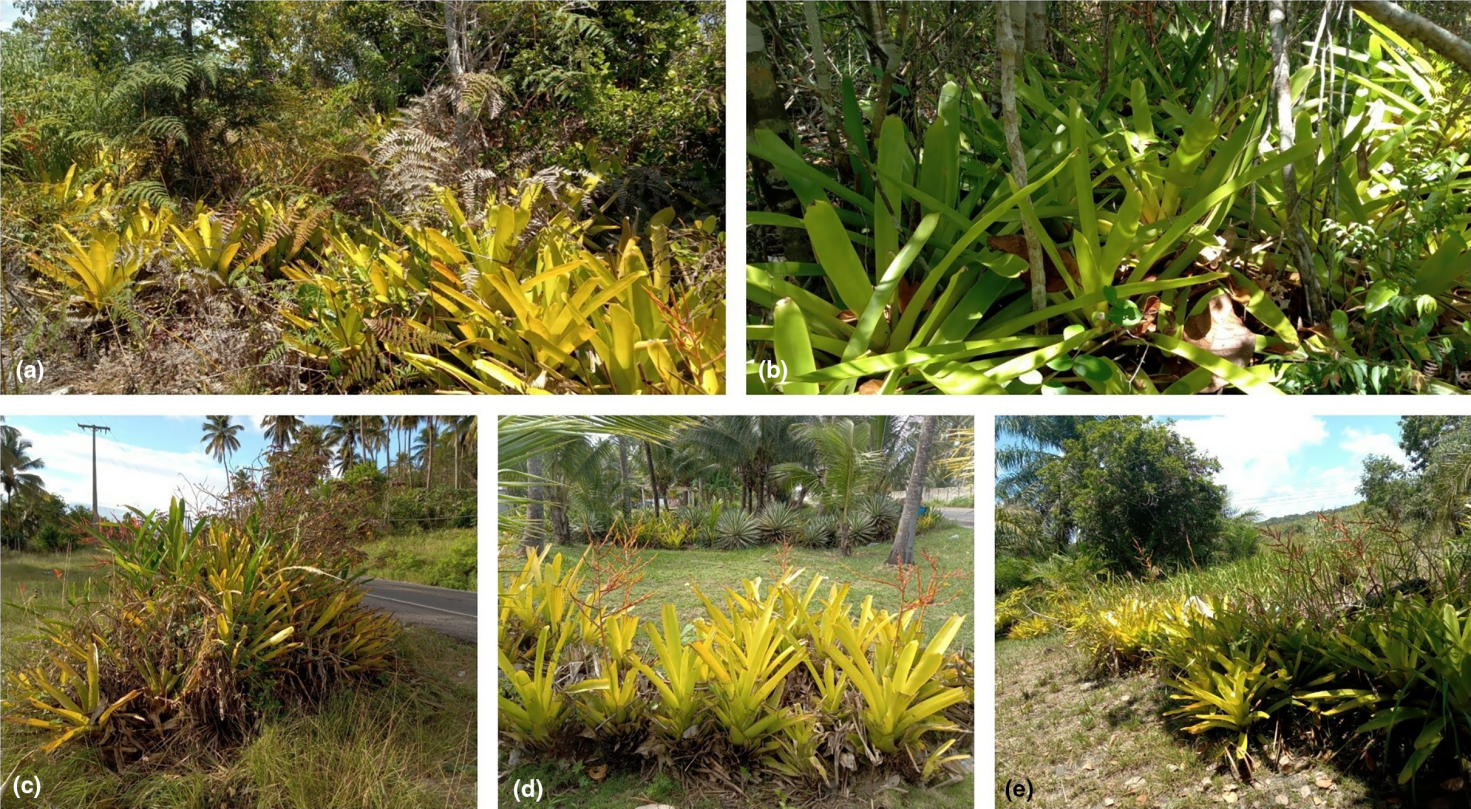
Breeding sites sampled in the municipality of Ilhéus, state of Bahia, northeastern Brazil. Sites a (−15.0844° S, −39.0080° W) and b (−15.0844° S, −39.0133° W) were 2 km far from highways with car traffic (natural environments and no human noise), while sites c (−14.9398° S, −39.0126 °W), d (−14.9289° S, −39.0177° W) and e (−15.0866° S, −38.9987° W) were 2–10 m close to highways with vehicle traffic (noise pollution), the distance from locations c and d to e was 15 km.

Field activities were carried out during the month of October 2021 (the peak in breeding activity of the species in the region) at five sampling sites in the municipality of Ilhéus, Bahia, Brazil. All areas have bromeliads used as reproductive sites by *Phyllodytes luteolus*. Three sites with the presence of vehicle traffic noise were located next to the BA‐001 highway (between 3 and 10 m away from the highway), in the stretches between Olivença and Acuípe villages, in the municipality of Ilhéus. The BA‐001 highway runs along the coast of the state of Bahia and its southern end begins in the city of Mucuri while the northern end is located on Itaparica Island, in the Todos‐os‐Santos Bay of the capital Salvador. The stretch of road on which it was selected based on the presence of individuals of *P. luteolus*, at night periods the frequency of vehicles decreased markedly when compared to daytime periods, but there was the presence of vehicles in passages. There is no data available from government agencies regarding the flow of vehicles on this stretch of the highway, but during our field activities we found that the greatest traffic occurs between 5 and 7 pm. Two other sampling sites without the presence of vehicle traffic noise were defined at a minimum distance of two kilometers from the BA‐001 highway (Carr & Fahrig, 2001) (Figure 2). In these sites, there was no other source of anthropic noise, eventually few noises from other species that actively communicate acoustically.

The sampling sites were defined as Natural (no traffic noise) or Highways (with traffic noise). For each recorded individual, we obtained the social situation in which the male was inserted, being: with chorus (four or more conspecific individuals in vocalization activity within a 3‐m radius – CC) or without chorus (less than four conspecific individuals vocalizing within a radius of three meters – SC).

Males of *Phyllodytes luteolus* in vocalization activity were found through visual and auditory searches between 19:00 and 24:00 h. The sampling points form extensive areas with many bromeliads individuals and, therefore, a large population of *P. luteolus*. To avoid recording the same individual in vocalization activity more than once, we started the search for *P. luteolus* males on different sides of the sampling points, thus ensuring that pseudoreplication biases did not occur, since the individuals were not marked. After viewing the male, the advertisement calls were recorded using a Sennheiser ME66 directional microphone coupled to a Marantz PMD660 hand recorder at a distance of 0.5 m and 1.5 m in height from the vocalizing male. As *P. luteolus* males have a long silent interval between calls, each male was recorded for 10 min, in order to obtain as many calls as possible. The recordings were obtained in WAV format, 44 kHz sampling rate and 16‐bit resolution. The bioacoustic analyses were performed with the Raven Pro 1.6 software, using the following configuration for the spectrogram: type = hann, FFT = 512, overlap = 50%. Three to five calls were analyzed for each individual. The acoustic parameters extracted were: call duration (s), number of notes, number of pulses per call, pulse duration (s), rise time (s), interval between calls (s), repetition rate (calls/min) and dominant frequency (Hz). The acoustic terminologies followed Köhler et al. (2017). The sound pressure level (SPL dB re 20 μPa; intensity) was obtained for three to five advertisement calls from each individual using a Minipa MSL‐1301 decibel meter (range: 30–130 dB; 125 ms, fast, precision 1.5 dB, curve dBA) positioned directly in front of and 0.5 m away from the male in vocalization activity.

Shortly after the recordings were finished, the males were captured for measurement of the snout‐vent length (SVL) with a Western caliper (accuracy of 0.1 mm). Air temperature and humidity were recorded using a thermo‐hygrometer (TechnoLine WS 9440, 0°C precision). The variables were obtained for all recorded individuals. The recordings are deposited in the Fonoteca Neotropical Jacques Vielliard (catalog number; 0058292 to 0058297).

The noise level present in the environment was measured from the calling site of each male recorded using a Minipa MSL‐1301 decibel meter (range: 30–130 dB; 125 ms, fast, precision 1.5 dB, curve dBA), positioned 1.5 m above the ground. The decibel meter was positioned in the four cardinal directions (north, south, east, west), and for each direction three values of sound pressure level (SPL; intensity) were obtained. Every 20 seconds, we recorded the highest value (three records per minute), so on for each direction. Subsequently, an average of all recorded values was extracted to represent the environmental noise present during the vocalization activity of males. To describe the frequency noise of the traffic during the calling activity of the calling males, we also obtained frequency values (peak and bandwidth 90% frequency ‐ Hz) from the recordings of individuals near the road. In the recordings of males from natural environments, the frequency values of the recordings were not measured because they were close to zero due to the absence of noise.

To verify if the environments close to the highway had higher environmental noise than natural environments, we performed a t‐test using the average SPL obtained from the calling site of each male. To test whether anthropic (hypothesis 1) and conspecifics (hypothesis 2) noise alter the acoustic parameters (response variables) of the advertisement calls of *Phyllodytes luteolus*, we performed multiple regressions using the type of environment (with and without traffic noise) and situation (with and without chorus) as explanatory variables. Since body size and air temperature can influence anurans calls (Gambale & Bastos, 2014; Morais et al., 2012; Wells, 2007), these two factors were used as covariates in the models. We tested the multicollinearity of the model's independent variables through a variance inflation factor (VIF) test. We considered variables with VIF values greater than 3 to be collinear. The VIF did not indicate collinearity between the variables (environment = 2680, situation = 1063, SVL = 2520, and temperature = 1088). We evaluated the assumptions of normal distribution of residuals and homogeneity of variances by graphs of quantiles and residuals as a function of adjusted values (Zuur et al., 2009), respectively. Acoustic parameters that did not meet the assumptions (call duration, number of pulses and interval between calls) were logarithmized.

To test significant differences in the set of acoustic parameters between the environments (with and without traffic noise), a multivariate analysis of permutational variance (PERMANOVA, Anderson, 2001) based on a Euclidean distance matrix was used. Before this analysis, we performed a Pearson correlation between the acoustic parameters and removed the highly correlated ones (number of notes, number of pulses, rise time and interval between songs). To verify the homogeneity of the variances, we used the PERMDISP (Homogeneity of Multivariate Dispersion) (Anderson, 2004). For all tests, we assigned a significance level lower than 5%. The analyses were performed using the “vegan”, “lme4” and “car” packages in the R software version 4.1.1 (R Core Team, 2016).

## 3 RESULTS

**FIGURE 3:**
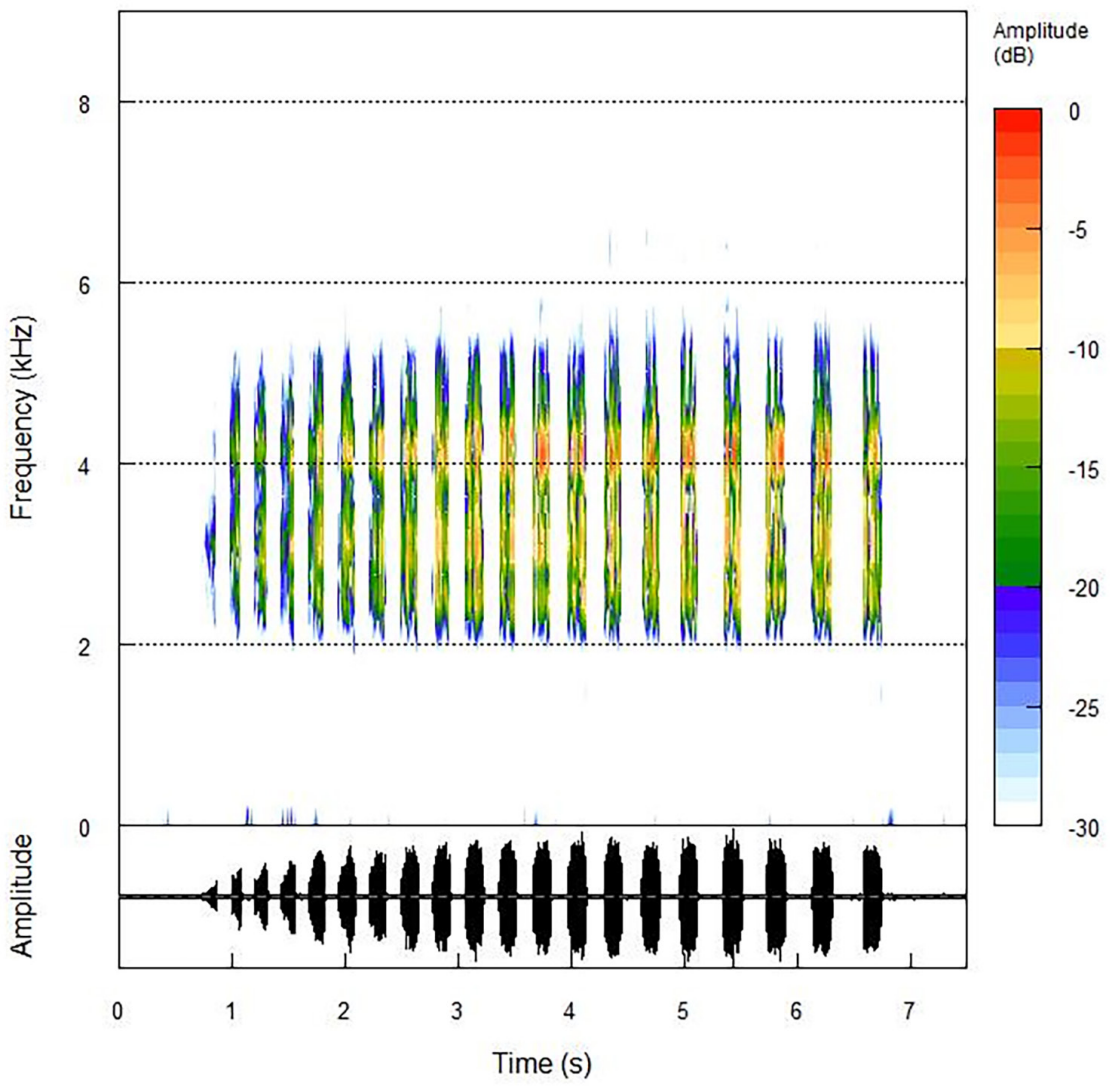
Advertisement call structure of *Phyllodytes luteolus* (Wied‐Neuwied, 1821), spectrogram (above) and oscillogram (below) (air temperature = 25°C; body size = 23.7 mm), calling in a natural environment in the municipality of Ilhéus, Bahia, Brazil.

**FIGURE 4:**
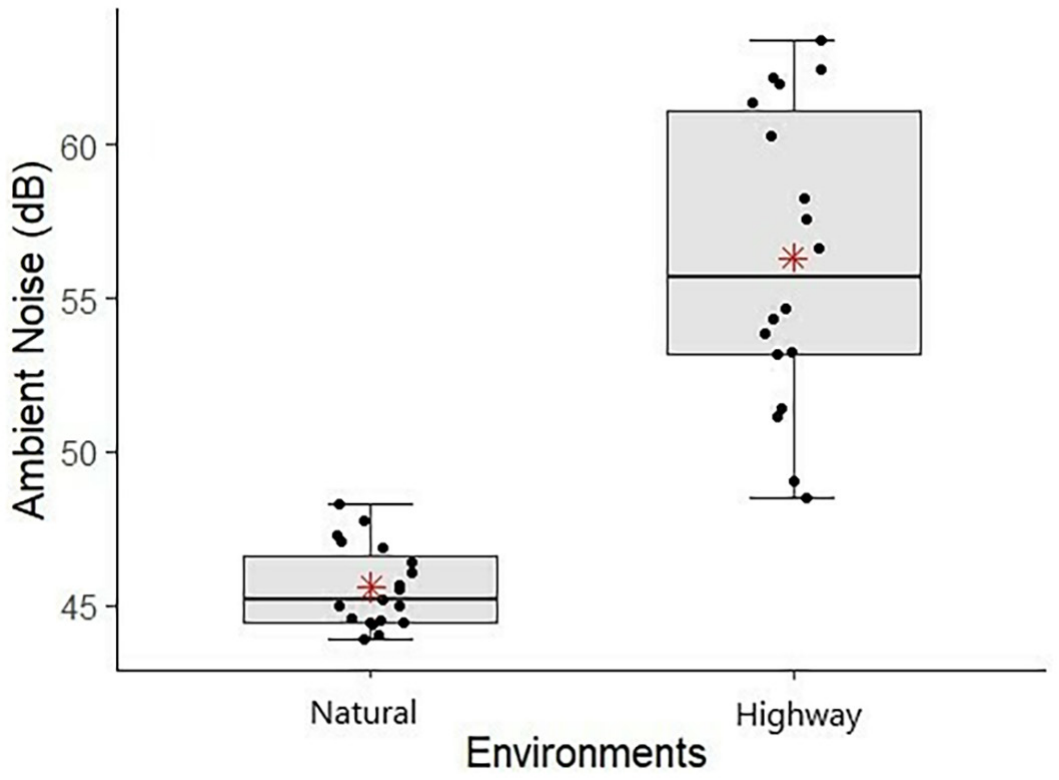
Boxplot representing the differences in intensity (dB; sound pressure level) recorded between environments (Natural and Highway), in the municipality of Ilhéus, Bahia, Brazil. The boxplots show the median (middle line), first and third quartiles (upper and lower bounds of the box), the points presented in the boxplot represent each noise value in decibels for each individual and the extreme lines indicate the non‐outlier range. The red points represent the mean values.

Recordings of 38 males of *Phyllodytes luteolus* were obtained, 18 in road environments (nine individuals in situation with chorus and nine individuals in situation without chorus) and 20 in natural environments (six individuals in situation with chorus and 14 individuals in situation without chorus). The advertisement call structure is represented in Figure 3. The mean values and standard deviation of each acoustic parameter of the advertisement call of *P. luteolus* between each environment and situation are represented in Table 1. The environmental noise differed significantly between the two environments (*t* = −9.45, df = 37, *p* < .05). Higher noise levels were found in sites close to highways (average = 56.2 dB) compared to natural areas (45.6 dB) (Figure 4). Peak and bandwidth 90% frequencies of recordings from highway environments averaged 129.199 Hz (86.133–947.461 Hz) and 1143.281 Hz (258.398–4048.242 Hz).

**TABLE 1:** Mean and standard deviation values of each acoustic parameter of the advertisement call of *Phyllodytes luteolus* between environments and situations in which males were exposed.

Acoustic parameters	Environments			
	Highway (*n* = 19)		Natural (*n* = 20)	
	With chorus (*n* = 9)	Without chorus (*n* = 10)	With chorus (*n* = 6)	Without chorus (*n* = 14)
Dominant frequency (kHz)	3473.3–345.7	3460.4–389.8	3874.5–298	3889.9–314.1
Call duration (s)	5.554–0.803	5.713–1.712	5.751–1.736	5.127–0.719
Rise time (s)	3.020–0.630	3.162–1.170	2.902–0.822	3.365–0.881
Interval between calls (s)	75–31.7	69.3–18.4	79.9–15	78.8–27.9
Notes number	18.7–2.948	17.9–2.514	19.3–2.658	18.2–3.445
Pulse number	806.9–239.3	1016.7–483.7	884.6–71.2	771.5–154.9
Pulse duration (s)	0.0028–0.0005	0.0026–0.0007	0.0024–0.0001	0.0025–0.0004
Intensity (dB SPL)	71.1–4.22	69.9–4.06	71.7–7.20	70.8–6.14
Advertisement call rate (calls/5 min)	3.6–1.414	3.9–1.100	3.3–1.032	3.7–1.138
Advertisement call total	7.5–3.16	7.1–1.72	6.6–1.21	7.1–2.21

**FIGURE 5:**
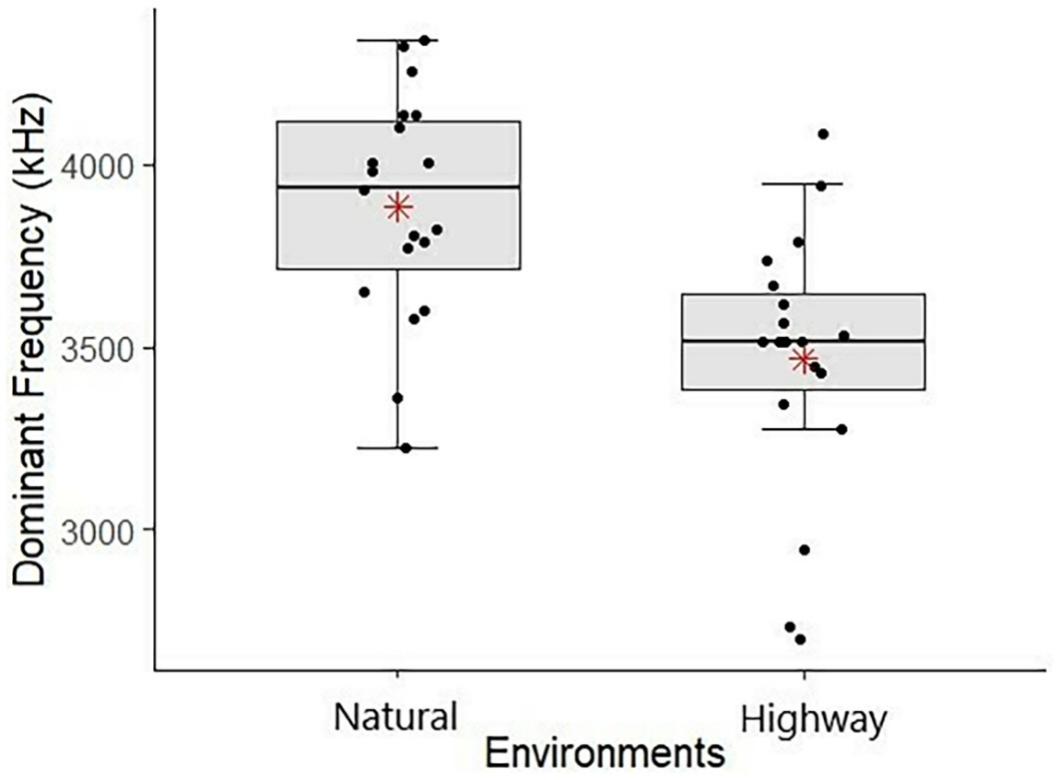
Boxplot representing the differences in the dominant frequency of the advertisement call of *Phyllodytes luteolus* between environments (Natural and Highway) in the municipality of Ilhéus, Bahia, Brazil. The boxplots show the median (middle line), first and third quartiles (upper and lower bounds of the box), and the extreme lines indicate the non‐outlier range. The red points represent the mean values.

**FIGURE 6:**
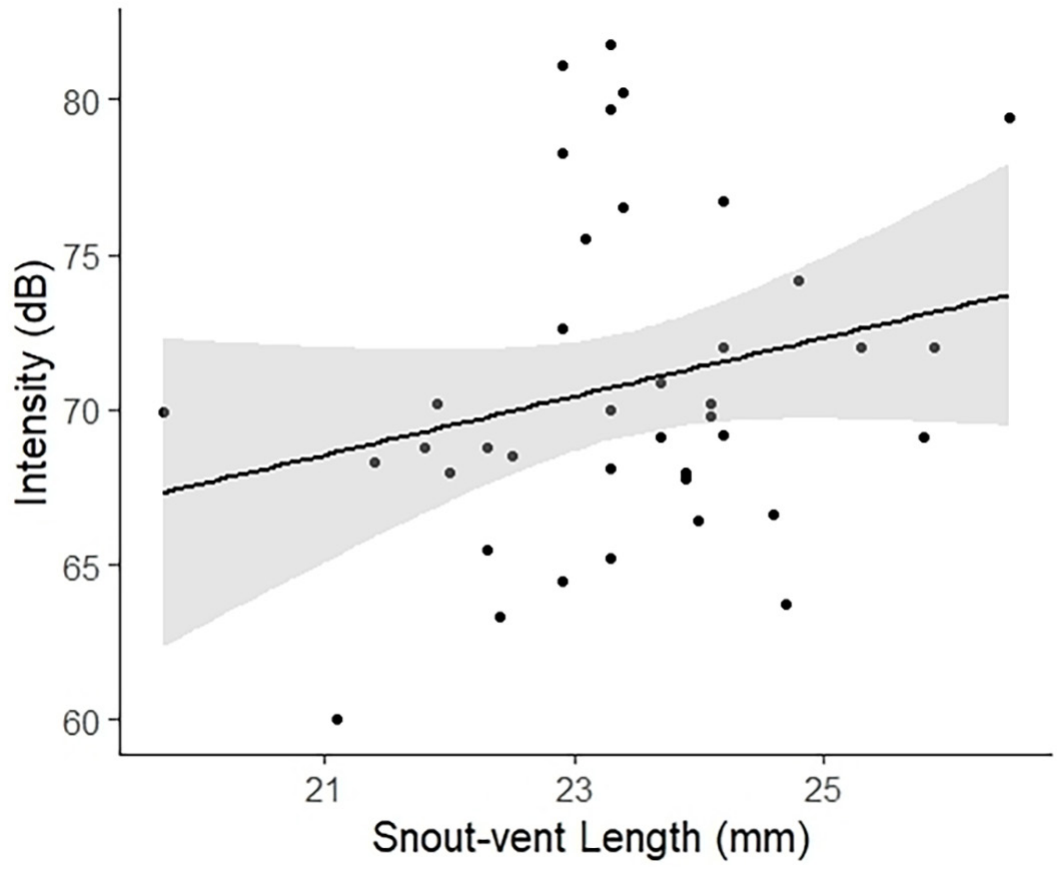
Positive relationship between advertisement call intensity (dB; sound pressure level) and body size of males of *Phyllodytes luteolus*. The line represents the estimated value from the linear regression analysis.

The dominant frequency of the advertisement call differed significantly between the two environments (*E* = −358.4, df = 33, *p* = .048), so that males from the sites in the natural areas (without road noise) showed higher values of the dominant frequency (Figure 5). No other acoustic parameter showed a significant difference between the environments in relation to the presence of traffic or situation (with or without chorus) (Table 2). Additionally, we found a significant positive relationship between the intensity of the calls and the snout‐vent length (*E* = 3.302, df = 33, *p* = .003) (Table 2), that is, the larger the individual the higher the signal intensity (Figure 6). Considering the set of acoustic parameters, there was no influence of the environment or the situation on the acoustic behavior of *P. luteolus* (*F* = 0.005, df = 36, *p* = .942).

**TABLE 2:** Results of multiple regression analysis between the acoustic parameters of the advertisement call of *Phyllodytes luteolus* on the independent variables recorded in the municipality of Ilhéus, Bahia, Brazil.

Acoustic parameters	Independent variables	Coefficients	Standard error	*p*
Dominant frequency (kHz)	Environments	−358.458	174.900	**.048**
	Situation	−7.457	119.583	.950
	SVL	−33.771	74.628	.653
	Temperature	−2.960	49.657	.952
Call duration (s)	Environments	0.469	0.635	.465
	Situation	−0.170	0.434	.698
	SVL	−0.114	0.271	.675
	Temperature	−0.170	0.180	.352
Rise Time (s)	Environments	−0.207	0.459	.655
	Situation	0.249	0.314	.432
	SVL	0.079	0.196	.686
	Temperature	0.169	0.130	.203
Interval between calls (s)	Environments	−16.908	12.708	.192
	Situation	−1.433	8.688	.870
	SVL	4.442	5.422	.419
	Temperature	−0.028	3.608	.994
Notes number	Environments	−0.013	0.116	.906
	Situation	−0.056	0.079	.477
	SVL	−0.004	0.049	.922
	Temperature	0.005	0.033	.856
Pulse number	Environments	86.76	0.597	.554
	Situation	45.42	99.43	.650
	SVL	15.09	62.05	.809
	Temperature	74.40	41.29	.080
Pulse duration (s)	Environments	1.077	2.761	.416
	Situation	−1.824	1.888	.924
	SVL	6.487	1.178	.586
	Temperature	−8.021	7.838	.314
Intensity (dB SPL)	Environments	−27.156	34.011	.430
	Situation	−0.453	1.685	.789
	SVL	3.302	1.051	**.003**
	Temperature	0.722	0.700	.309
Call rate (call/5 min)	Environments	0.299	0.266	.261
	Situation	0.061	0.179	.731
	SVL	−0.123	0.114	.282
	Temperature	−0.062	0.073	.393
Advertisement call total	Environments	1.194	1.074	.274
	Situation	−0.110	0.734	.881
	SVL	−0.403	0.458	.385
	Temperature	−0.443	0.305	.155

*Note*: In bold, significant *p*‐values (<.05).

## 4 DISCUSSION

Males of *Phyllodytes luteolus* presented advertisement calls with a higher dominant frequency in natural environments when compared to highway environments, and there is no evidence that chorus noise alters the acoustic parameters of this species. Therefore, *P. luteolus* does not seem to be affected by traffic noise, which may explain its presence in anthropized areas. In addition, we found a positive relationship between advertisement call intensity and male body size.

Contrary to what was expected by our hypothesis (1), males of *Phyllodytes luteolus* from natural areas presented higher dominant frequencies of the advertisement call compared to individuals from environments with road noise. Although some anuran species can increase the dominant frequency of calls to avoid overlapping with the noise frequency (e.g., *Scinax nasicus*, Leon et al., 2019; *Litoria ewingii*, Parris et al., 2009; *Rana clamitans* and *Rana pipiens*, Cunnington & Fahrig, 2010, and *Amolops torrents*, Zhao et al., 2018), others can decrease the dominant frequency (e.g., *Boana bischoffi*, Caorsi et al., 2017). In an experimental study, Caorsi et al. (2017) observed that *Boana leptolineata* did not change the dominant frequency when exposed to different levels of anthropic noise. Like *B. leptolineata*, males of *Phyllodytes luteolu*s have calls with an average dominant frequency (3.46 kHz) above the mean energy concentration of road noise (below 1 kHz) (Cunnington & Fahrig, 2010; Warren et al., 2006). Therefore, species that have calls with higher frequencies have little or no acoustic overlap with anthropic noise. In addition, the frequency of ambient noise in highway areas was very low and may not represent sufficient noise to disrupt *P. luteolus* vocalizations. The dominant frequency of the call is usually a static property (little intra‐individual variation) as it is related to morphological characteristics of males (Gerhardt, 1991; Köhler et al., 2017), serving as an indicator of the quality of males (Bastos et al., 2011; Gingras et al., 2013), being an important parameter for the sexual selection system of a population (specific recognition and mate selection by females). Thus, dominant frequency values may be stabilized across populations (Andreani et al., 2021; Friedl, 2006). Future studies should investigate possible causes for the difference in call frequency of different anuran populations, especially those exposed to anthropic noise.

The advertisement call of *P. luteolus* has a narrow bandwidth in which the energy distributions around the dominant frequency are short. Some studies suggest that acoustic signals with higher bandwidths may be less detectable against noise since the signal energy is spread over a wide range of frequencies (Parris et al., 2009; Warren et al., 2006), therefore *P. luteolus* would have an advantage in noisier environments. Many altered habitats present a constant noise pattern in certain frequency bands and for a long period of time, acting as a selection pressure (Warren et al., 2006). In this sense, more studies need to be carried out to assess how different types of noise, and/or even anthropogenic substrate‐borne vibrations (e.g., Caorsi et al., 2019) can influence the reproductive behavior of anuran amphibians.

There was no influence of road noise or situation (with or without chorus) on the temporal parameters of the advertisement call of *P. luteolus*, also not corroborating our hypothesis (II). The advertisement call of *P. luteolus* has a long duration (around 5 s) and a series of identical notes without frequency modulation. The temporal structure of the call can influence the active space of the signal when propagated in the environment, increasing the fidelity of the transmitted information, and, therefore, decreasing the probability of acoustic overlap (Ey & Fischer, 2009; Warren et al., 2006), an attribute that is advantageous in environments with high noise levels. In general, the social context in which males are inserted in the reproductive site influences the change in call parameters (Bosch & De La Riva, 2004; Gerhardt et al., 2000; Toledo et al., 2015). Furthermore, males of *P. luteolus* begin to vocalize after the period of greatest road traffic (between 5 and 7 pm), and therefore, the lower flow of vehicles at night may not represent a source of noise that modifies the acoustic parameters of the species. It is necessary to investigate whether other factors are more important to determine changes in the acoustic parameters of *P. luteolus*, such as the density of conspecific individuals in the chorus. Experimental studies on the immediate response of males are important for a better understanding of the behavioral strategies adopted by *P. luteolus* on the balance of costs and benefits determined by changes in acoustic parameters when they are subjected to different noise levels.

We found a positive relationship between advertisement call intensity and male body size. In general, morphological characteristics of males can determine the temporal parameters of the call (Köhler et al., 2017; Wells, 2007). For example, larger males have larger lungs and, therefore, greater energy reserves, being able to emit calls with greater intensity (Wells, 2007; Wells & Schwartz, 2006). Possibly, even in noisy scenarios, larger males are able to increase the intensities of their calls to avoid signal masking and promote greater signal detection, localization and discrimination (Halfwerk et al., 2016; Yi & Sheridan, 2019). Therefore, the intensity of the call is an important parameter for sexual selection (Gerhardt & Huber, 2002; Wells, 2007) since the sounds with higher intensities can propagate over long distances, potentially attracting a greater number of females and thus obtaining greater reproductive success (Bastos et al., 2011; Kime et al., 2000; Penna & Solís, 1998).

The Yellow Heart‐tongued Frog (*Phyllodytes luteolus*) has advantageous acoustic characteristics in conditions where anthropogenic noise is present. Therefore, even in noisy environments, individuals of this species manage to recognize and discriminate in the conspecific chorus. This fact may explain the successful occupation of areas outside its original range, having already been reported as an invasive species (Forti et al., 2017). Invasive species may impose limits on reproductive success and survival of other frog species that live in the same habitat. For example, males of *P. luteolus* calling in the same frequency range as the species *Ischnocnema* sp. The acoustic noise caused by the vocalization of an invasive species can harm the acoustic communication system in native species as it can influence the ability of females to locate males in the reproductive environment (Forti et al., 2017). This is the first study evaluating the influence of sound noise on a bromeliad anuran. Future studies may increase our knowledge on the effects of anthropogenic sound and serve as a subsidy for conservation actions, especially those aimed at acoustic monitoring in noisy environments.

## 5 CONCLUSION

This work investigated the aspects of the environmental condition, social context and morphological factors on the acoustic parameters of the Yellow Heart‐tongued Frog (*Phyllodytes luteolus*) advertisement call when exposed to anthropic road noise. The results show that the dominant frequency of calls is higher in environments without traffic noise. In addition, we also found that larger males can produce signals with greater intensity. As the emission of calls is the main form of communication in anurans, we concluded that *P. luteolus* presented characteristics in the structure of the advertisement call that may represent advantages in noisy anthropic environments. Future studies are important to investigate how different sources of noise can affect in different ways the acoustic communication and population dynamics of organisms that use the emission of acoustic signals to communicate.

## AUTHOR CONTRIBUTIONS

**Leonardo Marques de Abreu:** Conceptualization (lead); data curation (lead); investigation (lead); methodology (lead); visualization (lead); writing – original draft (lead). **Vinicius Guerra:** Formal analysis (equal); methodology (equal); writing – review and editing (equal). **Mirco Solé:** Project administration (equal); resources (equal); supervision (equal); writing – review and editing (equal).

## CONFLICT OF INTEREST STATEMENT

The authors declare that they have no known competing financial interests or personal relationships that could have appeared to influence the work reported in this paper.

### OPEN RESEARCH BADGES

This article has earned Open Data, Open Materials and Preregistered Research Design badges. Data, materials and the preregistered design and analysis plan are available at https://datadryad.org/stash/share/oNiODkagphjaPL4UJ30irvI‐LEKIzRmeuOcT00xUhvI.

## Data Availability

The recordings made in this study have been deposited in the Jacques Villiard Neotropical Phonotheque (https://www2.ib.unicamp.br/fnjv/) and are available under record numbers 58292 to 58297. The data used in this study are available at https://doi.org/10.5061/dryad.3bk3j9krb.
